# PD-1/PD-L1抑制剂治疗NSCLC脑转移的免疫机制与临床研究进展

**DOI:** 10.3779/j.issn.1009-3419.2020.102.31

**Published:** 2020-11-20

**Authors:** 佳敏 盛, 晓晴 俞, 晖 李, 云 范

**Affiliations:** 1 325035 温州，温州医科大学 Wenzhou Medical University, Wenzhou 325035, China; 2 310022 杭州，中国科学院肿瘤与基础医学研究所 Institute of Cancer and Basic Medicine (ICBM), Chinese Academy of Sciences, Hangzhou 310022, China; 3 310022 杭州，中国科学院大学附属肿瘤医院肿瘤内科 Department of Medical Oncology, Cancer Hospital of the University of Chinese Academy of Sciences, Hangzhou 310022, China; 4 310022 杭州，浙江省肿瘤医院肿瘤内科 Department of Medical Oncology, Zhejiang Cancer Hospital, Hangzhou 310022, China

**Keywords:** 肺肿瘤, 脑转移, 免疫治疗, PD-1/PD-L1, Lung neoplasms, Brain metastasis, Immunotherapy, PD-1/PD-L1

## Abstract

脑转移是非小细胞肺癌(non-small cell lung cancer, NSCLC)的常见并发症，预后较差。近年来，免疫检查点抑制剂将恶性肿瘤的治疗带入新纪元。程序性死亡因子-1(programmed death-1, PD-1)/程序性死亡因子配体-1(programmed death ligand 1, PD-L1)抑制剂通过激活自身免疫系统，产生抗肿瘤效应。PD-1/PD-L1抑制剂在NSCLC脑转移治疗中初露锋芒，但其确切疗效及最佳治疗模式仍有待进一步研究。本文针对NSCLC脑转移灶的免疫微环境，PD-1/PD-L1抑制剂在颅内的作用机制及其临床研究现状进行综述。

## Abstract (en)

Brain metastasis (BM) is a common complication in non-small cell lung cancer (NSCLC), which associates with poor prognosis. Recently, immune checkpoint inhibitors (ICIs) has revolutionized the treatment of tumors. Programmed death-1 (PD-1)/programmed death ligand 1 (PD-L1) inhibitors could produce antitumor effect by activating the autoimmune system. The immunotherapy has already show to have a promising outcome for NSCLC patients with BM, while its specific curative effect and the most ideal mode of the treatment remain to be explored. Here we reviewed the tumor microenvironment (TME) in BM lesions and summarized the role of PD-1/PD-L1 inhibitors in cerebral and its current status in clinical studies.

非小细胞肺癌(non-small cell lung cancer, NSCLC)易发生脑转移，约10%-20%的NSCLC患者初诊时即可发生脑转移，随着疾病进展，脑转移的发生率可高达30%-50%^[1-3]^。近年来，基于诊断水平的不断提高及NSCLC患者生存时间的显著延长，脑转移的发生率随之升高^[4]^。NSCLC患者一旦发生脑转移，总体预后欠佳。手术和放疗等局部治疗手段虽可缓解症状，但在患者总生存时间(overall survival, OS)的延长上作用有限。因大部分化疗药物不能较好地透过血脑屏障(blood brain barrier, BBB)，化疗仍然不是NSCLC脑转移患者主要的治疗手段。靶向治疗的兴起为驱动基因阳性的NSCLC脑转移患者提供了新的选择，以奥希替尼、阿来替尼等为代表的酪氨酸激酶抑制剂能有效透过BBB，显著延长驱动基因阳性NSCLC脑转移患者的生存时间，已成为该类患者的标准治疗^[3, 5]^。但驱动基因阴性的NSCLC脑转移患者，亟需新的治疗手段。

免疫治疗是肿瘤治疗的“变革者”，以程序性死亡因子-1(programmed death-1, PD-1)/程序性死亡因子配体-1(programmed death ligand 1, PD-L1)抑制剂为代表的免疫检查点抑制剂(immune checkpoint inhibitors, ICIs)在晚期NSCLC开展了一系列Ⅲ期随机对照研究，显示出惊人疗效。晚期NSCLC患者的5年生存率由化疗时代的5%提高至16%-23%^[6]^。一些小样本的回顾性研究和真实世界研究^[7, 8]^显示，ICIs对颅内转移灶也能表现出良好的抗肿瘤活性。本文就脑转移灶的免疫微环境、ICIs对颅内转移灶的作用机制及其临床研究现状进行综述。

## 1 脑转移灶的免疫微环境

由于血脑屏障和血脊髓屏障对外周免疫细胞的阻隔，同时缺乏与外周淋巴结的有效淋巴引流，大脑一直被认为是免疫豁免器官，一旦肿瘤细胞转移至颅内，则可躲避免疫系统的监视和杀伤。近年来随着脑膜淋巴管的发现，以及血脑屏障的完整性会被脑转移灶破坏的理论得到证实，目前普遍认为免疫细胞可从外周循环浸润至颅内^[9-11]^。血液来源的免疫细胞与颅内驻留细胞(如星形胶质细胞、小胶质细胞和神经元等)共同构成了一个复杂、动态的肿瘤微环境(tumor microenvironment, TME)^[12]^。

根据“种子-土壤”学说，TME对肿瘤细胞在颅内生长和转移有重要作用，而脑转移灶TME总体上是处于免疫抑制状态。颅内TME中不同的刺激可使小胶质细胞分化成M1型和M2型：M1型小胶质细胞可增加信号转导与转录激活因子1(signal transducer and activator of transcription 1, STAT1)的表达，产生炎性细胞因子，抑制肿瘤生长; M2型小胶质细胞会释放免疫抑制因子，如白细胞介素(interleukin, IL)-10和转化生长因子β(transforming growth factor β, TGF-β)，增加STAT3的表达，抑制抗原呈递，从而促进肿瘤生长^[13, 14]^。小胶质细胞/肿瘤相关巨噬细胞(tumor associated macrophages, TAMs)亦可协助肿瘤细胞穿过血脑屏障，促进肿瘤细胞生长^[12]^。星型胶质细胞与小胶质细胞一样亦具有两面性。星形胶质细胞生理状态下维持微环境稳态，早期与肿瘤细胞接触时，可诱导可溶性凋亡相关因子配体(soluble apoptosis-related factor ligand, sFasl)引起肿瘤细胞凋亡，从而阻止肿瘤的转移定植^[15]^。但肿瘤细胞-星形胶质细胞之间的缝隙连接可激活星形胶质细胞，使其促进肿瘤细胞增殖，保护肿瘤细胞免受化疗杀伤^[16]^。此外，星形胶质细胞外泌体中的microRNA可导致颅内微环境中肿瘤细胞第10号染色体同源丢失性磷酸酶基因和张力蛋白(phosphate and tension homology deleted on chromsome ten, PTEN)的表达可逆性下调，继而促进脑转移瘤的生长^[17]^。Henrik等^[18]^发现由于星形胶质细胞-小胶质细胞复杂的相互作用，分泌大量免疫抑制因子(如IL-10、TGF-β)，产生免疫抑制微环境，继而使T细胞活性减弱。

既然脑转移灶的TME处于免疫抑制状态，那么肺癌原发病灶和脑转移灶的TME之间是否存在差异？Kim等^[19]^对比研究了20例NSCLC脑转移患者的肺部原发灶和脑转移灶，发现CD3^+^肿瘤浸润淋巴细胞(tumor infiltrates lymphocytes, TILs)、CD4^+^ TILs、CD8^+^ TILs及FoxP^+^TILs的浸润丰度和PD-L1表达无明显统计差异，但脑转移灶中PD-1^+^ TILs的浸润丰度显著降低; 而在其中15例肺腺癌脑转移灶中，FoxP^+^ TIL及PD-1^+^ TILs的浸润丰度较肺部原发病灶有明显降低，差异有统计学意义。Teglasi等^[20]^在61例肺腺癌脑转移患者的肺部原发灶和脑转移灶中发现，肿瘤细胞PD-L1的表达在脑转移灶与原发病灶中存在显著正相关，且不受化疗或激素治疗的影响。与肺部原发灶相比，脑转移灶中PD-L1的表达和TILs的浸润减少。另有研究^[21]^通过对比肺部原发灶与脑转移灶的免疫相关基因表达谱发现，在脑转移灶中树突状细胞成熟、Th1和白细胞外渗信号通路受到抑制，促炎细胞黏附分子血管细胞粘附蛋白1(vascular cell adhesion protein 1, VCAM1)表达显著下调，而巨噬细胞浸润显著升高。这一异质性在一定程度上可以解释PD-1/PD-L1抑制剂在颅内外病灶中的疗效差异。肺部原发灶与脑转移灶的PD-L1表达是否可能有时空异质性？Mansfield等^[22]^对73例NSCLC脑转移患者的肺部原发灶和脑转移灶进行配对比较后发现肺部原发灶和脑转移灶在时间和空间上均存在差异：在空间上，在73例患者中，10例(14%, κ=0.71)患者的PD-L1表达在配对病灶中不一致。相比于肿瘤组织，配对病灶的免疫细胞PD-L1表达不一致为19例(26%, κ=0.38)。在时间上，间隔6个月以上发生脑转移的患者配对病灶的PD-L1表达不一致性高于间隔小于6个月的患者，但是差异没有统计学意义。免疫细胞PD-L1表达不一致性的趋势相同，也不存在统计学差异。

## 2 PD-1/PD-L1抑制剂治疗脑转移灶的可能机制

尽管有基础研究显示，脑转移灶的肿瘤微环境相对处于免疫抑制状态，但多项研究及一些个案报道证明ICIs治疗脑转移灶仍然具有一定疗效。那么，ICIs作用于颅内病灶的可能机制是什么？有以下几个问题一直引起广泛关注：①PD-1/PD-L1抑制剂是否能够透过血脑屏障？一些临床前动物数据证实ICIs可穿透血脑屏障，但ICIs不直接作用于肿瘤，而是激活免疫系统来对抗肿瘤^[23]^。因此，药物对血脑屏障的渗透性并不是至关重要的; ②活化的T淋巴细胞能否浸润脑部病灶？抗原递呈细胞处理肿瘤细胞抗原活化后，激活T细胞及NK细胞，两者均可通过脑膜淋巴系统进入脑转移瘤内发挥杀伤肿瘤的作用^[24]^。有研究^[25, 26]^报道，ICIs可显著促进T淋巴细胞(主要是CD8^+^ T淋巴细胞)由颅外向颅内募集(颅内T淋巴细胞数量可增长为原有的14倍)，进而杀伤颅内肿瘤细胞，产生有效的抗肿瘤效应; ③脑转移灶的免疫环境是否影响疗效？有研究^[19, 27, 28]^报道肺癌脑转移灶中的肿瘤细胞PD-L1表达水平和TILs的浸润程度与预后相关。Teglasi等^[27]^在208例肺腺癌脑转移患者中发现瘤周存在单核细胞环提示预后较好，而PD-1和PD-L1的低表达与较好的预后相关。但Kim等^[19]^在15例肺腺癌脑转移人群中发现颅内CD3^+^ TIL及PD-1^+^ TILs的高浸润与较差的预后相关，而PD-1^+^ TILs在脑转移瘤中的低浸润与颅内病灶免疫治疗响应较差密切相关。Berghoff等^[28]^在116例脑转移样本中(包括61例NSCLC)发现CD3^+^ TILs、CD8^+^ TILs和CD45RO+ TILs的浸润丰度与生存预后成正相关，且CD8^+^ TILs的浸润丰度与术前颅脑磁共振显像中瘤周水肿程度呈正相关。综上所述，免疫治疗使NSCLC脑转移患者获益，从机制上是可行的。

## 3 PD-1/PD-L1抑制剂治疗NSCLC脑转移的疗效

NSCLC脑转移在临床症状、影像表现、疾病进程及治疗方案的选择上均存在明显的异质性。到目前为止，尚无PD-1/PD-L1抑制剂针对NSCLC脑转移患者开展的前瞻性Ⅲ期随机对照研究。目前报道的有关PD-1/PD-L1抑制剂治疗NSCLC脑转移的疗效数据主要来自3个方面：①针对晚期NSCLC的大型Ⅲ期随机对照研究中的脑转移亚组分析; ②针对脑转移开展的前瞻性、小样本的单臂研究; ③真实世界的临床研究数据。

Nivolumab、Pembrolizumab及Atezolizumab在晚期NSCLC的二线及一线治疗中开展了多项大型、Ⅲ期随机对照研究，旨在比较免疫单药/联合化疗与标准化疗之间的疗效与不良反应。多项研究结果证明PD-1/PD-L1抑制剂与标准化疗相比具有明显的优效性及良好的耐受性，从而奠定了PD-1/PD-L1抑制剂在晚期NSCLC中的一线/二线标准治疗的地位。这些Ⅲ期随机对照临床研究中有部分研究允许无症状或经治且稳定的脑转移患者入组，我们将对其中的脑转移亚组进行具体的阐述与分析。

### 3.1 PD-1/PD-L1抑制剂单药治疗NSCLC脑转移

#### 3.1.1 晚期NSCLC Ⅲ期临床研究的脑转移亚组

PD-1/PD-L1抑制剂单药治疗NSCLC脑转移的疗效一直是大家关心的问题，因为单药能够直观地观察到药物的疗效。在上述大型Ⅲ期随机对照研究中，脑转移入组病例数相对较多的有OAK研究。OAK研究^[7]^共纳入850例经治晚期NSCLC患者，其中Atezolizumab组和多西他赛组分别纳入61例和62例无症状小脑幕上转移的NSCLC脑转移患者。研究结果显示，Atezolizumab组和多西他赛组脑转移患者的中位OS分别为16.0个月和11.9个月(HR=0.74, 95%CI: 0.49-1.13, *P*=0.163, 3);脑转移患者出现脑部新发转移灶的中位时间，Atezolizumab组未达到，多西他赛组为9.5个月(HR=0.38, 95%CI: 0.16-0.91, *P*=0.023, 9)。在安全性方面，使用Atezolizumab治疗的脑转移患者仅2例(3%)出现3级神经相关的不良事件(adverse event, AE)，未观察到4级/5级神经相关AE。尽管在脑转移亚组，Atezolizumab组相比多西他赛组的中位OS未达到统计学差异，但对颅内新发病灶有一定的预防作用; Atezolizumab在脑转移患者的治疗中安全性可控。

Nivolumab单药在晚期NSCLC的二线治疗中有3项主要临床研究：CheckMate-017研究、CheckMate-057研究和CheckMate-063研究，但这3项临床研究中入组的脑转移患者数均偏少。Goldman等^[8]^将这3项研究中的脑转移亚组进行荟萃分析，共纳入脑转移患者88例，结果发现Nivolumab组较化疗组中位OS有获益趋势，分别为8.4个月(95%CI: 5.0-11.6)和6.2个月(95%CI: 4.4-9.2);6个月颅内新病灶发生率有降低(13% *vs* 17%)。该荟萃分析提示，Nivolumab治疗脑转移患者有OS获益趋势，能延缓颅内新病灶发生。

这两项数据基本说明了目前晚期NSCLC大型临床研究中有关脑转移亚组的情况，由于这些研究对脑转移的入组有非常严格的限制，入组的脑转移患者数量十分有限，且都是颅内肿瘤负荷较小的稳定脑转移患者，并不能很好地代表NSCLC脑转移的整体人群，所以无法完整反映PD-1/PD-L1抑制剂治疗脑转移人群的疗效，但基本可以观察到PD-1/PD-L1抑制剂对脑转移的疗效。

#### 3.1.2 小样本、单臂的前瞻性研究

目前针对NSCLC脑转移开展的前瞻性研究尚处于Ⅰ期/Ⅱ期，样本量较小。Goldberg等^[29]^在一项Pembrolizumab治疗黑色素瘤及NSCLC脑转移的Ⅱ期临床研究中，纳入39例伴有至少1个5 mm-20 mm脑转移病灶的NSCLC患者，分成队列1包括34例PD-L1阳性的NSCLC患者和队列2包括5例PD-L1表达阴性的NSCLC患者。队列1的研究结果显示，颅内客观缓解率(intracranial objective response rate, iORR)为29.4%，中位OS为8.9个月(95%CI: 6.6-29.7)，颅内中位无进展生存期(intracranial progression free survival, iPFS)为10.7个月(95%CI：6.6-未达到); 31%的脑转移患者存活超过2年(95%CI: 19%-51%)。此外，研究未出现大于1级且与治疗相关的神经系统AE。

FIR研究^[30]^是一项旨在评估Atezolizumab治疗晚期NSCLC患者疗效和安全性的Ⅱ期临床研究，该研究中一个队列纳入了13例经治的无症状NSCLC脑转移患者，应用Atezolizumab治疗的客观缓解率(objective response rate, ORR)为23%，中位OS为6.8个月(95%CI: 3.2-19.5)，中位无进展生存期(progression free survival, PFS)为4.3个月(95%CI: 1.1-16.2)。CheckMate-012研究^[8]^是一项Ⅰ期多队列研究，其中一个队列纳入了12例初治的无症状NSCLC脑转移患者，接受单药Nivolumab治疗的ORR为16.7%，疾病控制率(disease control rate, DCR)为16.7%，中位OS为8.0个月(95%CI: 1.38-15.50)，中位PFS为1.6个月(95%CI: 0.92-2.50)。从这些小样本、前瞻性研究中可以观察到，PD-1/PD-L1抑制剂治疗NSCLC脑转移患者，颅内病灶的近期疗效与颅外病灶类似，但PFS及OS似乎较短，这种现象是因为小样本研究的偏差还是其他可能，值得进一步研究观察。

#### 3.1.3 真实世界的回顾性研究

除了大型临床研究的脑转移亚组及小样本的前瞻性研究，来自真实世界的回顾性研究数据是这方面的有力补充。一项意大利Nivolumab扩展入组计划(expanded access programme, EAP)研究分别对经治晚期的肺鳞癌患者(*n*=371)和非鳞NSCLC患者(*n*=1, 588)展开研究，其中纳入了37例经治的无症状肺鳞癌脑转移患者^[31]^，Nivolumab治疗脑转移患者的ORR为19%，DCR为49%，中位OS为5.8个月(95%CI: 1.9-9.8)，中位PFS为4.9个月(95%CI: 2.7-7.1)，脑转移患者和总人群的1年OS率分别为35%和39%，1年PFS率分别为31%和27%。而在409例经治无症状非鳞NSCLC脑转移患者的亚组分析中^[32]^，ORR为17%，DCR为39%。脑转移人群和总人群的中位PFS分别为3.0个月(95%CI: 2.7-3.3)和3.0个月(95%CI: 2.9-3.1)，1年PFS率分别为20%和22%，中位OS分别为8.6个月(95%CI: 6.4-10.8)和11.3个月(95%CI: 10.2-12.4)，1年OS率分别为43%和48%。上述EAP研究结果表明，Nivolumab对NSCLC脑转移患者的疗效与总人群基本一致。

此外，Hendriks等^[33]^收集了1, 025例接受PD-1/PD-L1抑制剂治疗的晚期NSCLC患者，其中脑转移患者255例(39.2%为活动性脑转移患者，27.4%接受皮质类固醇治疗)。伴脑转移患者较不伴脑转移患者的中位OS和PFS较短(8.6个月*vs* 11.4个月，*P*=0.035;1.7个月*vs* 2.1个月，*P*=0.009)。活动性脑转移患者iORR为27.3%，比稳定性脑转移更易发生颅内疾病进展(progressive disease, PD)(54.2% *vs* 30%, *P* < 0.001)。在脑转移亚组的多变量分析中，使用皮质类固醇与较差的生存预后有关(HR=2.37)，而稳定的脑转移(HR=0.62)和更高的疾病相关性预后分级(diagnosis-specific graded prognostic assessment, ds-GPA)(HR=0.48-0.52)有较好的生存获益。

这些回顾性研究在很大程度上肯定了PD-1/PD-L1抑制剂在NSCLC脑转移患者中的治疗作用，但由于回顾性研究本身的局限性，并不能全面回答PD-1/PD-L1抑制剂在脑转移治疗中的一些关键问题，例如确切的疗效、真正获益的人群、最佳的治疗时机及治疗方案等。

### 3.2 PD-1/PD-L1抑制剂联合化疗

肿瘤细胞利用多种机制发生免疫逃逸，免疫与化疗强强联合不仅能增强宿主免疫系统对肿瘤细胞的识别和消除，还可以改善肿瘤微环境的免疫抑制状态^[34]^。

KEYNOTE-189研究^[35]^是一项随机双盲、Ⅲ期临床研究，旨在评估Pembrolizumab联合培美曲塞和铂类一线治疗转移性非鳞NSCLC患者的疗效。该研究共纳入108例EGFR/ALK阴性的非鳞NSCLC脑转移患者，其中Pembrolizumab联合化疗组和单纯化疗组分别为73例和35例。研究结果显示，Pembrolizumab联合化疗相较单纯化疗，可显著改善NSCLC脑转移患者的中位OS，分别为19.2个月(95%CI: 15.0-25.9)和7.5个月(95%CI: 4.6-10.0)。

Powell等^[36]^荟萃分析了KEYNOTE-021研究、KEYNOTE-189研究和KEYNOTE-407研究中的脑转移亚组，研究共纳入脑转移患者171例，研究结果显示，Pembrolizumab联合化疗比单纯化疗可改善OS(HR=0.48, 95%CI: 0.32-0.70)和PFS(HR=0.44, 95%CI: 0.31-0.62)，而且ORR更高(39.0% *vs* 19.7%)，中位缓解持续时间(duration of response, DOR)更长(11.3个月*vs* 6.8个月)。综合上述研究提示，免疫联合化疗可改善NSCLC脑转移患者的生存预后。

### 3.3 PD-1/PD-L1抑制剂联合放疗

放疗能够诱导肿瘤抗原释放，改善肿瘤免疫微环境，引发“远隔效应”，与免疫治疗产生协同抗肿瘤反应^[37-40]^。

一些回顾性研究结果提示，ICIs与放疗联合治疗脑转移可以增强疗效，而且似乎二者同步进行相比序贯和单用放疗疗效更佳。一项回顾性研究^[41]^分析了260例接受立体定向放射治疗(stereotactic radiosurgery, SRS)的脑转移患者(包括157例NSCLC患者，70例黑色素瘤患者和33例肾癌患者)。研究结果显示，SRS同步联合免疫治疗组较非同步联合治疗组及单纯放疗组明显延长OS(24.7个月*vs* 14.5个月*vs* 12.9个月，*P*=0.006)。Ahmed等^[42]^回顾性分析了17例NSCLC脑转移患者，共有49个脑转移灶。其中22个病灶为放疗后序贯免疫治疗，13个病灶为放疗同步免疫治疗，14个病灶为免疫治疗后序贯放疗。研究结果显示，在免疫治疗前或同步接受放疗的脑转移患者，6个月iORR为57%，而免疫后序贯放疗的脑转移患者则为0%(*P*=0.05)。上述两项研究提示，免疫治疗同步接受放疗对于脑转移患者可能具有更好的疗效。

此外，有两项研究观察了免疫联合放疗的安全性。一项回顾性研究^[43]^收集了54例NSCLC脑转移患者，其中免疫联合SRS组37例，单纯SRS组17例。该研究结果显示，两组放射性坏死或瘤内出血发生率无明显增加(5.9% *vs* 2.9%, *P*=0.99)，瘤周水肿发生率亦无显著差异(11.1% *vs* 21.7%, *P*=0.162)。研究提示免疫联合放疗治疗NSCLC脑转移患者耐受性较好，不良反应无明显增加。但Martin等^[44]^回顾性分析了480例接受SRS的脑转移患者(包括294例NSCLC、145例黑色素瘤和41例肾细胞瘤)，其中115例(包括38例NSCLC)脑转移患者接受ICIs联合治疗(药物包括Ipilimumab、Pembrolizumab和Nivolumab)。研究结果显示，免疫联合放疗较单纯放疗的放射性脑坏死发生率增高(23/115, 20% *vs* 25/365, 6.8%)，且与免疫治疗相关(*P*=0.004)。研究提示，免疫联合放疗应警惕放射性脑坏死的发生。

上述回顾性研究提示，免疫联合放疗显示出较好的生存获益，总体安全性较好，但应警惕放射性脑坏死的发生。关于联合治疗中放疗的最佳时机以及如何与其他治疗手段进行有效整合等仍需进一步探究。

## 4 存在的问题

综上所述不难发现，有关PD-1/PD-L1抑制剂治疗NSCLC脑转移的临床数据仍然非常粗糙和有限。尽管我们引用了不少PD-1/PD-L1抑制剂治疗NSCLC的大型随机对照研究的脑转移亚组数据，但是，这些研究对脑转移患者的入组有着非常严格的限制; 均排除了活动性或有症状的脑转移患者及所有的脑膜转移患者; 有的研究要求脑部病灶必须经过脑部放疗且病灶稳定。显而易见，这些入组的脑转移患者与真实世界的脑转移人群存在较大的差距。其次，在这些临床研究中最终能够成功入组的脑转移患者数量非常少，这在很大程度上影响了统计的效能，导致尽管可以观察到研究组有较好的OS延长，但两组最终并没有差异。再者，到目前为止，最有指导价值的前瞻性的临床研究仍然非常缺乏，哪怕是样本量稍大一点的单臂Ⅱ期研究。

## 5 总结与展望

PD-1/PD-L1抑制剂单药治疗在NSCLC脑转移患者中已经初步观察到疗效，但这种治疗手段是否适合所有的脑转移人群，需要进一步研究; 因为，已经有研究提示似乎活动性脑转移患者并不能从单纯的免疫治疗中获益。免疫联合化疗及抗血管生成治疗是晚期NSCLC的标准治疗，在脑转移人群亦有较好的疗效数据。放疗在NSCLC脑转移的治疗中占据有非常重要的地位; 因此，如何将PD-1/PD-L1抑制剂与放疗、化疗及抗血管生成治疗进行有机结合，是未来的研究关键。进一步探索脑转移病灶的免疫微环境，研究预测疗效的相关生物标记物，将有助于PD-1/PD-L1抑制剂更加有效地用于NSCLC脑转移患者的治疗。
